# Rapid face orienting in infants and school-age children with and without autism: Exploring measurement invariance in eye-tracking

**DOI:** 10.1371/journal.pone.0202875

**Published:** 2018-08-28

**Authors:** Kirsten A. Dalrymple, Natalie Wall, Michael Spezio, Heather C. Hazlett, Joseph Piven, Jed T. Elison

**Affiliations:** 1 Institute of Child Development, University of Minnesota, Minneapolis, Minnesota, United States of America; 2 Psychology and Neuroscience, Scripps College, Claremont, California, United States of America; 3 Institute for Systems Neuroscience, University Medical Center, Eppendorf, Hamburg, Germany; 4 Carolina Institute for Developmental Disabilities, University of North Carolina at Chapel Hill, North Carolina, United States of America; Universite de Bretagne Occidentale, FRANCE

## Abstract

Questions concerning the ontogenetic stability of autism have recently received increased attention as long-term longitudinal studies have appeared in the literature. Most experimental measures are designed for specific ages and functioning levels, yet developing experimental tasks appropriate for a wide range of ages and functioning levels is critical for future long-term longitudinal studies, and treatment studies implemented at different ages. Accordingly, we designed an eye-tracking task to measure preferential orienting to facial features and implemented it with groups of participants with varying levels of functioning: infants, and school-age children with and without autism. All groups fixated eyes first, revealing an early and stable orienting bias. This indicates common bias towards the eyes across participants regardless of age or diagnosis. We also demonstrate that this eye-tracking task can be used with diverse populations who range in age and cognitive functioning. Our developmental approach has conceptual implications for future work focused on task development and particularly new experimental measures that offer measurement equivalence across broad age ranges, intellectual functioning and verbal abilities.

## Introduction

Questions concerning ontogenetic stability and change in the autism spectrum disorder (ASD) phenotype have received increased attention in recent years. This is due in large part to the appearance of long-term longitudinal studies. For example, studies have called the universal stability of an ASD diagnosis into question by identifying individuals who had an initial diagnosis of autism, yet later function within normal limits [1]. A long-term longitudinal study of individuals diagnosed with autism at 2-years separated the individuals into three outcome groups, based on distinct trajectories on measures of verbal and non-verbal IQ, social adaptation, social communication, repetitive sensory motor behaviors, and insistence on sameness [2]. These authors postulated that longitudinal trajectories of behavioral profiles may represent meaningful phenotypes, which researchers could leverage to parse the heterogeneity of ASD for genetic studies. A series of reports from the *Pathways in ASD* group (www.asdpathways.ca) has also refuted the universal stability assumption, quantifying heterogeneity in longitudinal trajectories from initial preschool diagnosis into school-age [3–5]. These studies underscore the benefits of implementing longitudinal designs to track variability of the natural history of ASD, but also highlight the need for new experimental measures that offer measurement equivalence [6] across broad age ranges, intellectual functioning and verbal abilities.

A common domain of inquiry in individuals with ASD, which has proven useful as a measure of treatment response in young children [7], is face processing. In general, the face processing literature is hindered by small sample sizes, variability in methodological considerations (e.g. dynamic vs. static stimuli, task parameters, etc.), variability in age of assessments and the assumption that findings from one age group should generalize to other ages, and the inherent heterogeneity of the phenotype [8, 9]. That said, studies with sufficiently large samples to approximate the heterogeneity of autism have begun to appear [10]. Early studies suggested children with autism use mouth information more than controls in face recognition tasks, while controls tend to rely more on information from the eye region to perform the same tasks [11]. This latter finding has been replicated in various studies using free viewing of video clips of complex social scenes [12], identifying emotional expression from faces [13] and free viewing of static face stimuli [14]. Other evidence suggests that young children with autism may show equivalent looking at the eye region while gazing less at the mouth when presented with static stimuli [15] indicating that a preference for the mouth region may not be universal in ASD [16, 17]. However, little is known about long-term longitudinal changes in face processing (but see [18] for findings from the first 2 years).

One useful task for assessing face processing is the “bubbles” technique [19]. This technique involves revealing faces through randomly selected Gaussian windows (“bubbles”) that are superimposed onto the facial images underneath, to assess the visual information used by an observer to make a categorical decision about the face (e.g. male vs. female, afraid vs. happy, etc.). Gaussian window locations that covary with higher performance on the task are considered to contain the information that is more important for making the correct categorization on the given task. Using this technique combined with eye tracking technology, Spezio et al. [20] found no difference between a group of adults with high functioning autism and age and IQ matched controls in terms of accuracy, reaction time, or number of bubbles required to perform the task (determining whether a face showed fear or happiness), indicating that task performance can appear equivalent between groups. However, despite equivalent performance on the task, the bubbles methodology revealed that the ASD group made more use of the mouth and less use of the eyes than controls when making judgments of facial expression, a finding that extended to a subgroup of parents who showed aloof features of the broader autism phenotype [21]. This finding underscores the importance of assessing gaze location in addition to basic task performance (i.e. accuracy, reaction time), in the study of autism. In other words, it demonstrates that in orienting tasks, it is just as important to investigate the “how” as it is to investigate the “how well” [9].

**Fig 1:**
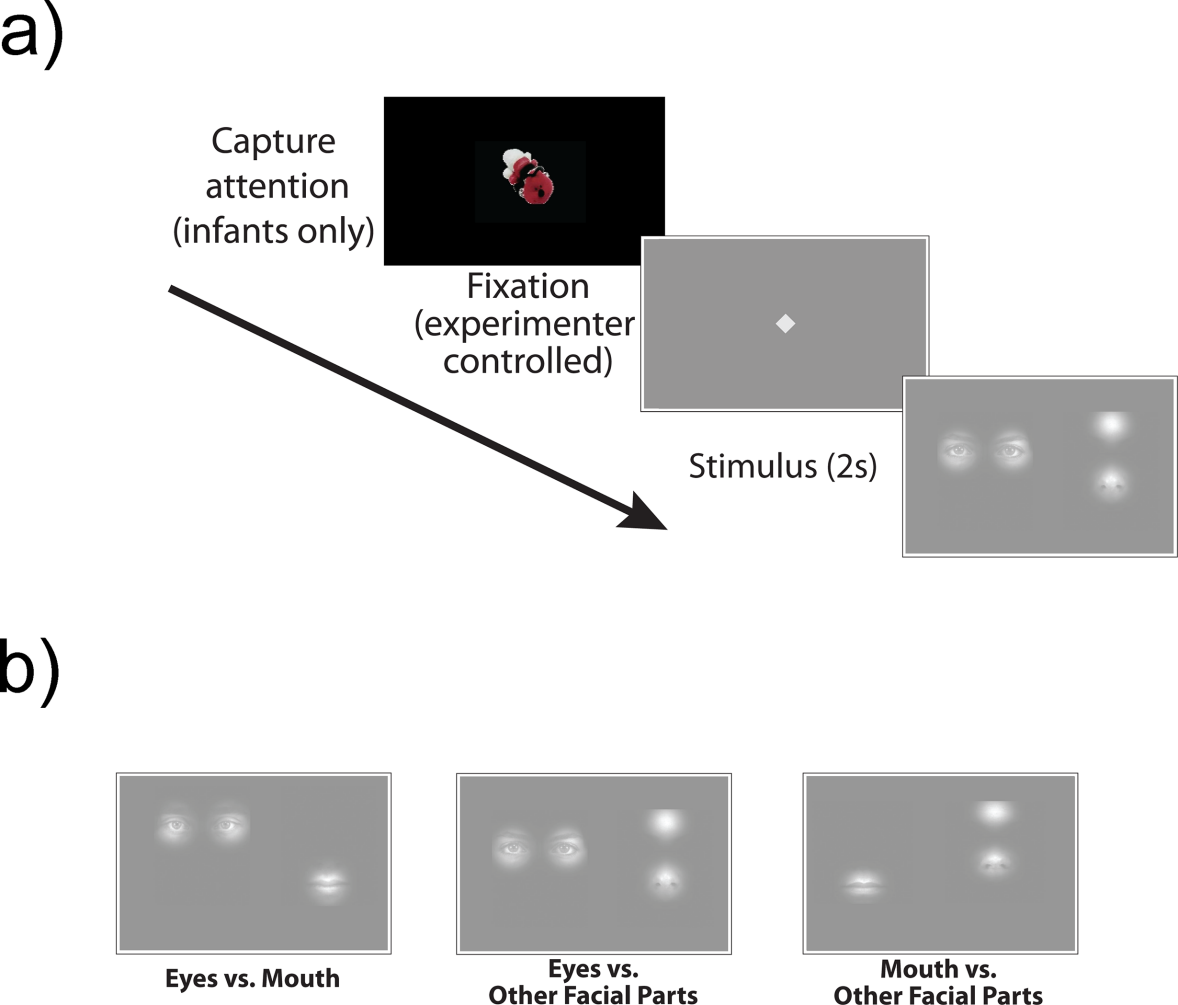
Bubbles paired visual comparison task. a) Task procedure. b) Various pairings of face parts: Eyes vs. Mouth, Eyes vs. Other Face Parts (OFPs), Mouth vs. OFPs. OFPs consisted of forehead, nose, and/or cheeks.

One limitation to the bubbles task is that some populations, such as infants and individuals with co-occurring intellectual disabilities, cannot provide responses (e.g. via key press) to complete the task as initially designed. Humphreys et al. [22] adapted the bubbles task for use with infants, creating a preferential looking task that paired a picture of the infant’s mother with a stranger to determine what information is used to identify a familiar face [22]. While this task provided a useful alternative for infant populations, it specifically tests identity processing and requires adaptations for each participant (i.e. using mother’s face). It is also unclear whether this version of the bubbles task would provide meaningful information across a broad age range. Hence, there is a need for face-orienting/processing tasks that can be implemented across a wide range of ages and functioning levels. Accordingly, we developed a paired visual comparison version of a modified bubbles task that can be used across ages and developmental abilities to investigate preferential orienting to facial features. Our task pairs two of three types of facial features: 1) eyes, 2) mouth, and 3) other facial parts (i.e. OFPs; nose, cheeks, forehead), revealed through static Gaussian windows (“bubbles”) (Fig 1).

We designed this task with three primary goals in mind: 1) to make a first attempt at designing a task that can be implemented across a broad range of ages and developmental abilities, and to test the task by 2) evaluating the stability of patterns of preferential orienting to the eye region of static faces from infancy to school-age, and 3) comparing orienting patterns in individuals with autism to two typically developing groups (i.e. infants and school-age children). Our results demonstrate an interesting equivalence between groups in terms of early interest in eyes, and ultimately suggest that this modified bubbles task is useful for measuring preferential orienting to specific facial features in infancy, childhood, and in atypical populations.

## Method

### Participants

Two groups of school-aged children were enrolled in this study as part of a follow-up longitudinal neuroimaging study initiated when the children were between 18 and 30 months of age [23]. As part of the follow-up assessment at school-age, typically developing children (TYP: n = 14 (5 female); age range = 7–13 years; M age = 10.1 years, SD = 1.7) and children with autism spectrum disorder (ASD: n = 21 (3 female); age range = 10–13 years; M age = 12.2 years, SD = 1.1) participated in structural neuroimaging, a comprehensive clinical assessment, and an experimental eye tracking battery at the Carolina Institute for Developmental Disabilities, University of North Carolina. Diagnoses of autism were made by experienced clinicians, and all cases exceeded diagnostic thresholds according to the Autism Diagnostic Observation Schedule—Generic [24] and the Autism Diagnostic Interview–Revised (ADI-R; [25]). Here, we report findings from performance on one of the eye tracking tasks. We also recruited a sample of typically developing infants (n = 37 (21 female); age range = 6–17 months; M age = 9.3 months, SD = 2.3), 17 of whom were assessed at the Carolina Institute for Developmental Disabilities and 20 of whom were assessed at the Institute of Child Development at the University of Minnesota. Repeated measures ANOVAs with factor of Group (Minnesota vs. Carolina) and Condition (Eyes-Mouth vs. Eyes-OFPs vs. Mouth-OFPs) indicated that these two groups of infants showed statistically equivalent patterns of performance on our two main metrics (see below, Analysis; all *ps*>0.05) and therefore formed one group.

These studies were approved by the Institutional Review Boards at the University of Minnesota and the University of North Carolina at Chapel Hill. Parental permission and informed consent was provided for all participants and informed assent was obtained for all school-age participants. This study was carried out in accordance with The Code of Ethics of the World Medical Association (Declaration of Helsinki).

### Stimuli and apparatus

Stimuli consisted of competing pairs of facial features displayed 3.7° (126 pixels) apart on either side of center-screen (Fig 1). Facial features were classified as Eyes, Mouth, or Other Facial Parts, which consisted of one or two facial parts: nose, cheeks, and/or forehead. Facial features were taken from 15 males and 15 females from the Karolinska database [26]. Facial features were displayed in “bubbles” [19, 22]. Briefly, the bubbles method reveals static facial features through Gaussian holes in a mask covering an underlying image (Fig 1A). Features were gray scale on a gray background and paired in 3 different conditions: Eyes vs. Mouth, Eyes vs. Other Facial Parts, and Mouth vs. Other Facial Parts (Fig 1B).

All facial features were revealed through round bubbles that were 6.5° x 6.5° (220 x 220 pixels) when viewed from a distance of 60 cm. Eyes were always paired and separated by 2.2° (75 pixels). OFPs appeared alone on 40% of OFP trials and paired with another face part on 60% of OFP trials. Cheeks were paired or alone. Noses were paired with foreheads. Because different faces were used to produce the stimuli, the exact placement of the features varied from trial to trial, as they would normally for faces. Trials began with a fixation shape (cross, circle, square, or diamond), which measured 1.25° wide (42 pixels). Stimuli were separated by 3.7° (126 pixels).

Stimuli were displayed on a 23-inch Acer wide-screen monitor and eyes were tracked using a Tobii X120 or TX300 eye tracker (Tobii Technology AB, www.tobii.com). The X120 eye tracker is a desk-mounted dark pupil tracking system that has a temporal resolution of 8.3 ms (sampling rate 120 Hz) and gaze accuracy of 0.4° according to the manufacturers. The X300 is a desk-mounted dark pupil tracking system that has a temporal resolution of 3 ms (sampling rate 300 Hz) and a gaze accuracy of 0.4° according to the manufacturers.

### Procedure

School-age participants sat 60-65cm from the display monitor. The experiment began with a 9-point calibration procedure in which participants fixated the centroid of dots that appeared on specified locations on the screen. After successful calibration (judged qualitatively by the trained examiner), participants heard instructions to look at the screen throughout the experiment. No further instructions were given. Trials began with a small shape at center-screen (cross-hair that subtended 1.25° (horizontal); Fig 1A) that remained on-screen until the experimenter determined that the participant was looking at the shape, at which time the experimenter initiated the presentation of the stimuli via keyboard press. When the key was pressed, a pair of competing bubbles facial features appeared for 2 s. The task consisted of 3 blocks of 30 trials (90 trials in total). Each competing pair of features (e.g. Eyes vs. Mouth) appeared 10 times per block. Each block had a different trial order. Analyses included data from participants who completed fewer than 3 blocks if they performed a sufficient number of trials to meet the inclusion criteria (see Analysis).

The procedure was similar for the infants, with the exception of the following: Parents held infants in their laps for the duration of the experiment. Calibration consisted of 5 dots presented at specified locations on the screen. During the experiment, some trials began with an attention-grabbing cartoon that moved and made noise at center-screen to attract the infants’ attention to the screen prior to the presentation of the fixation shape. The experimenter pressed a key to advance to the fixation shape when the infant was looking at the screen. Most infants attempted one block of 30 trials, but in effort to maximize data collection a small number of infants (n = 8) attempted two blocks. Because these infants had so few valid trials in their second block (only n = 5 had 25% or more valid trials), we opted for a standardized criterion of one block per infant. For infants who attempted two blocks, we analyzed the block that had the greater number of valid trials.

### Analysis

We first computed the average number of valid trials across all participants in all groups: Overall mean = 67.8% (SD = 27.8); TYP = 88.6% (SD = 19.7); ASD = 69.2% (SD = 30.7); Infants = 59.2% (SD = 24.8). Trials were considered valid if the participant spent at least 250 ms on-screen (250 ms was considered to be the minimum amount of time needed to reliably characterize an orienting response [27]). We then removed any participant with less than 25% valid trials (for infants, 25% valid trials within the included block). This criterion resulted from beginning with 1.5 standard deviations below the overall mean across all groups then rounding down to the more conservative value of 25% (22 trials out of 90 for school-age participants, and 8 out of 30 trials for infants). The final sample included n = 14 TYP (mean age = 10.1 years, SD = 1.7; 5 female), n = 18 ASD (mean age = 12.2 years, SD = 1.0; 2 female) and n = 32 Infants (all typically developing, mean age = 9.5 months, SD = 2.4; 20 female).

Areas Of Interest (AOIs) surrounded the facial features (left eye, right eye, mouth, and each OFP), using Tobii Studio (www.tobii.com). All AOIs were 6° x 7.6° when viewed from a distance of 60cm. We exported raw data indicating when gaze fell within an AOI. This produced a data point every 8.3 ms for recordings from the X120 and every 3 ms for the X300 and allowed us to aggregate gaze data on a given AOI. We chose not to implement a fixation algorithm as this decision is often linked to a specific level of cognitive processing under investigation, and we did not assume that ‘processing’ would be equivalent in the infants and school-aged children, per se. Additionally, in the absence of a specific a priori hypothesis linking level of processing to fixation time, the parameters selected for various fixation algorithms are often arbitrary [28]. We used the recording time stamps to calculate how much time was spent on-screen and on a given AOI. This also allowed us to determine which of the two competing facial features elicited the first look on each trial.

Using valid trials only (at least 250 ms on-screen), we computed a mean for each participant on two primary measures. The first measure is the “first feature”: which facial feature in a competing pair most often elicited the first look. Specifically, this measure consisted of the proportion of trials during which a given feature was gazed at first out of the total number of valid trials for that competing pair. The second measure is the proportion of gaze time: how much time was spent looking at each feature divided by the total looking time spent on-screen.

Each trial involved pairing two of the three facial feature types, resulting in missing values for the remaining feature type. To accommodate this design feature, we computed difference scores for each competing feature pair by subtracting the value associated with one feature from the value associated with the competing feature. Using these difference scores for “first feature” and proportion of gaze time, we performed a 3 x 3 Repeated Measures ANOVA with factors of Group (TYP x ASD x Infants) and Feature Pair (EyesVs.Mouth x EyesVs.OFPs x MouthVs.OFPs). For “Feature Pair”, a positive value indicates that the first feature listed was favored, and a negative score indicates that the second feature listed was favored. Main effects were investigated with Bonferroni paired comparisons. All *α* = 0.05, after correction.

Data are available for download in S1 File. The python script used to process the data is available in S2 File.

## Results

### Age and IQ

The TYP group was significantly younger than the ASD group, t(30) = 4.19, *p*<0.001, but there was no association between age and any of our dependent measures for either the TYP group (all *p*s>0.140) or the ASD group (all *p*s>0.260). Similarly, there was no association between age and any dependent variable for the Infants (all *p*s>0.220).

The Differential Ability Scales-II (2007, Pearson Education, Inc.) provided measures of IQ for the TYP and ASD groups. We computed full-scale IQ scores for all 14 of the children in the TYP group and 16 of the 18 children in the ASD group who contributed at least 25% valid trials (two children did not complete the verbal scales and therefore did not have full-scale IQ scores). The TYP group had a significantly higher mean IQ (mean = 115.9, SD = 15.8) than the ASD group (mean = 88.9, SD = 17.9), t(28) = 4.34, *p*<0.001. However, there was no association between IQ and any of our dependent measures for either the TYP group (all *p*s>0.180) or the ASD group (all *p*s>0.200).

### First feature

**Fig 2:**
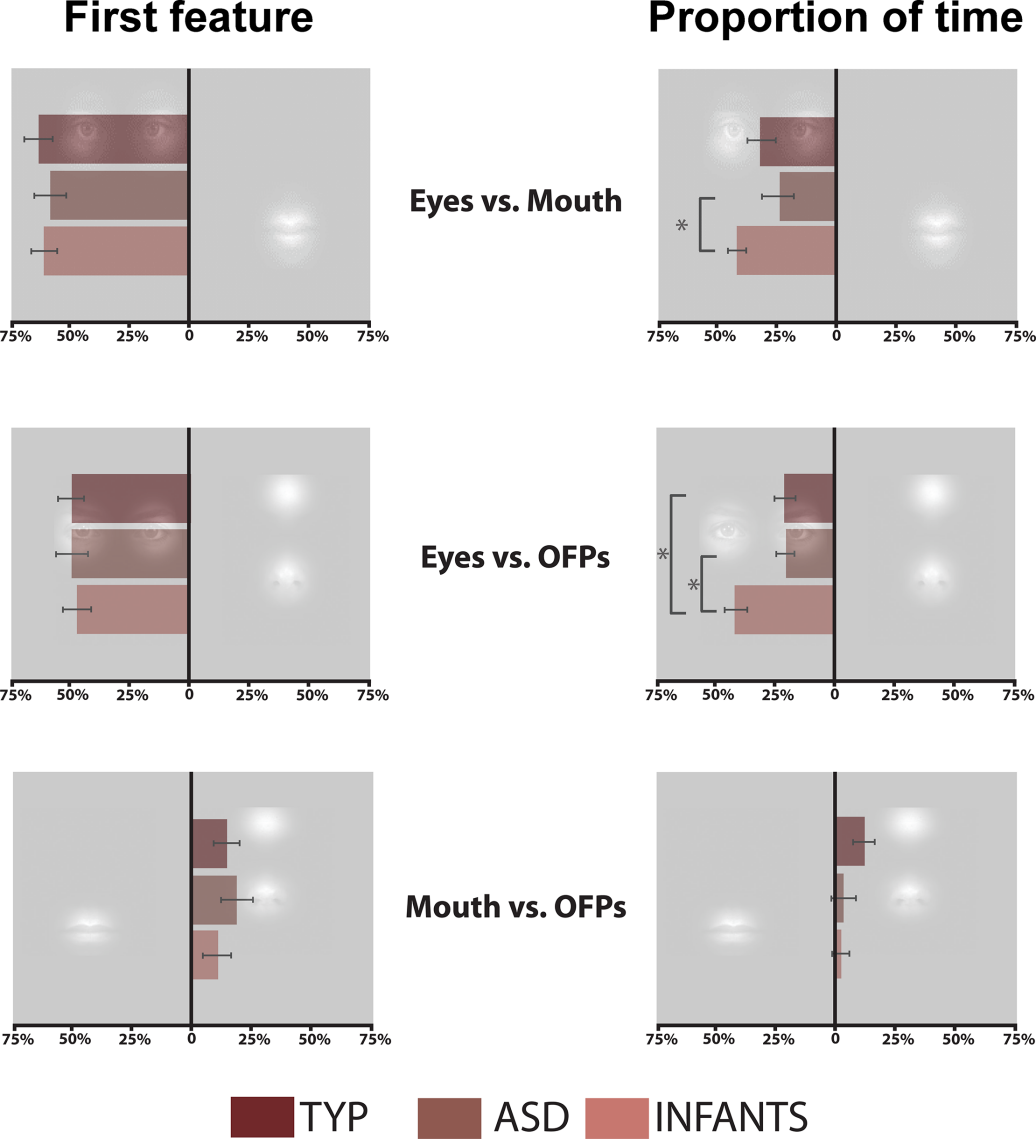
Results. a) Difference scores (in percent of trials) for which feature of a pair was looked at first. Difference scores were calculated by subtracting the percent of trials during which one feature was looked at first from the percent of trials during which the competing feature was looked at first. The direction of the bar indicates which feature of the pair was more frequently looked at first. b) Difference scores (in percent of total trial time) for time spent on competing features. Difference scores were calculated by subtracting the percent of the total trial time spent looking at one feature from the percent of total trial time spent looking at the competing feature. The direction of the bar indicates which feature of the pair had a higher dwell time measured as a percent of total trial time. Error bars represent +/- 1 standard error.

There was a main effect of Feature Pair, F(2, 61) = 77.36, *p*<0.001 (Fig 2A). Post-hoc Bonferroni paired comparisons indicated a lower difference score across groups for OFPs vs. Mouth (14.0%) than for both Eyes vs. Mouth (60.8%) (*p*<0.001) and Eyes vs. OFP (47.4%) (*p*<0.001). There was no main effect of Group (*p* = 0.871), nor was there a Group x Feature Pair interaction (*p* = 0.938). This indicates that all participants tended to look at the Eyes first when paired with the Mouth (75.9% vs. 15.1%) or OFPs (71.0% vs. 23.6%), but showed less of a tendency to look at the OFPs first when paired with the Mouth (52.5% vs. 38.5%).

In order to verify the statistical significance of the preference for the Eyes, we conducted 1-sample t-tests comparing the preference for Eyes (i.e. the difference score) to 0% for Eyes vs. Mouth and Eyes vs. OFPs, for each group. Indeed, for all groups, the preference for Eyes over other features was significantly greater than 0%, all *p*s < 0.001.

### Proportion of gaze time

For the measure of proportion of gaze time (Fig 2B), there was a main effect of Feature Pair, F(2, 61) = 92.7, *p*<0.001, Group, F(2,61) = 8.22, *p* = 0.001, and a Group x Feature Pair interaction, F(4, 61) = 3.37, *p* = 0.012. Post-hoc Bonferroni paired comparisons revealed a smaller difference between OFPs vs. Mouth (4.1%) than for both Eyes vs. Mouth (34.5%) (*p*<0.001) and Eyes vs. OFP (31.6%) (*p*<0.001) across all groups, and that Infants showed an overall greater difference score across all feature pairs than the TYP (*p* = 0.009) and ASD (*p* = 0.003) groups, who did not differ from one another.

Tests of simple effects revealed that the Group x Feature Pair interaction was driven by the Infants’ larger preference for Eyes over Mouth (46.4% vs. 4.5%), compared to the ASD group (41.6% vs. 18.5%), and for Eyes over OFPs (47.3% vs. 5.0%), compared to both the TYP (45.4% vs. 23.4%), and ASD (40.4% vs. 20.4%) groups (*p*s<0.05). The TYP and ASD groups did not differ from each other, nor did any of the groups differ in their degree of preference for OFPs over the Mouth: TYP 37.6% vs. 25.8%; ASD 27.8% vs. 25.5%; Infants 21.0% vs. 19.4% (*p*s>0.05).

As with the first feature measure, in order to verify the preference for the Eyes, we conducted 1-sample t-tests comparing the preference (i.e. difference score) to 0% for each Eyes vs. Mouth and Eyes vs. OFPs for each group. Again, for all groups, the preference for Eyes over the other features was significantly greater than 0% (all *p*s < 0.003).

## Discussion

The purpose of this study was to design and test a single task that can be implemented with a range of populations who have varying levels of age, verbal abilities, and cognitive functioning. We kept in mind three primary goals: 1) to make a first attempt at designing such a task, and to use the task to, 2) evaluate the stability of patterns of preferential orienting to the eye region of static faces from infancy to school-age, and 3) to compare orienting patterns in individuals with autism to two typically developing groups (i.e. infants and school-age children). Our approach was to design and test a paired visual comparison version of a bubbles face-orienting task [19, 22]. We monitored participants’ eye movements while they viewed pairs of facial features (eyes, mouth, and other facial parts (OFPs) such as nose, cheeks and forehead) that were briefly presented on opposite sides of fixation. Despite their inherent differences, all groups performed the task, and even had some interesting similarities in their looking behavior: all groups tended to look at the eyes first, regardless of the paired feature. Thus, across ages and developmental abilities, orienting to eyes is a behavior that emerges early and is stable over time. It also suggests a common attraction to particular social information that is present in typically developing children and preserved in autism. Differences between groups emerged when we examined dwell time. While all groups spent longer looking at the eyes than the mouth or OFPs, the tendency to dwell on eyes over OFPs was larger in infants compared to school-age children with and without autism. Infants also showed a larger preference for the eyes over the mouth than the ASD group. Thus, while early orienting is consistent across groups, dwell time shows differences between infants and school-age children. Interestingly, there were no differences between the typically developing children and children with ASD on either measure, suggesting a normative change in looking behavior with age, rather than with diagnosis.

The findings that both TYP and ASD groups look at the eyes first, and spend a larger proportion of time looking at them compared to the mouth, is inconsistent with some published findings that show that individuals with ASD tend to show the reverse pattern, a bias towards the mouth rather than the eyes (e.g. [11, 13, 14, 20, 29]). Although the literature on eyes vs. mouth looking in ASD is inconsistent (see, [16, 17]), one possible explanation for the difference between our findings and others is that our task is an orienting task rather than a classification or free viewing task where features appear in the context of a whole face or social scenes. Indeed, our findings are broadly consistent with findings from another rapid orienting task implemented with adults with autism [30]. Uniquely, our task puts facial features in direct competition with one another, with no other context or facial judgment requirements. This may lead to different behavioral patterns than when the participant is confronted with an entire face or with a task that requires goal-oriented cognitive processing of facial features. Another possible explanation for our findings is that our method of computing difference scores rather than comparing the time on the AOIs directly may have diluted differences between the ASD and TYP groups. To investigate this possibility, we calculated the average proportion of time spent on eyes and mouth, respectively, for each participant, ignoring what these AOIs were paired with on any given trial. Independent samples t-tests revealed no differences between the TYP to ASD children in terms of their mean proportion of time on the eyes (*p* = 0.299) or mouth (*p* = 0.972) throughout the task. Thus, this alternate analytic approach confirmed that the ASD children did indeed show a preference for the eyes over the mouth that was comparable to the TYP children.

The primary goal of the current study was to develop and test a procedure that can be implemented across a wide range of ages and developmental abilities, while maintaining measurement equivalence over time. The idea that an experimental task measures the same process across time and across heterogeneous samples is more often assumed than tested (but see [6]). As reliability and validity are not inherent properties of any measurement procedure, this type of examination promises to counter “detached validity” claims (c.f., [31]) that are pervasive in the field. We demonstrated that our task can be used successfully with infants and children, as well as special populations such as children with ASD. The task requires no goal-oriented instructions beyond passive viewing and can therefore be used with other, more severely impaired groups, such as individuals with below-average IQ, minimally verbal individuals, and individuals who are physically unable to make a key press or verbal response, allowing direct comparisons between groups. Thus, while this task may not be diagnostic in its current form, it illustrates an approach towards designing experimental tasks that can adequately test the assumption of ontogenetic stability in autism.

It is important to acknowledge some limitations of the current study. First, recruitment of the ASD group took place in the context of another study, and was therefore small, and not a truly random sample for the present study. That said, there were no existing characteristics of this group that would be expected to differentially affect their looking behavior during this task (we found no relationship between age or IQ and any of our dependent measures), and large sample sizes were not needed to achieve the primary goal of this study i.e. to demonstrate that it is possible to design a task that can be implemented across a broad range of ages and developmental abilities. We also wish to mention that even though we were able to achieve our goal with this task by demonstrating its feasibility, the final sample sizes for the school-age children would have ideally been larger to better represent the heterogeneity of the ASD and typical development. This task provides only a single measure of a given phenomenon and it would be beneficial to have a second task that measures the same behavior to provide converging evidence for orienting behaviors in these groups. Also, while the task presented here provides a simple, easy to use measure of basic looking behavior, it would be useful to design an alternate task with greater ecological validity to compare outcomes.

Another methodological issue worth discussing is data inclusion. We used a minimum valid trial criterion of 250 ms, meaning that trials were only included in the analysis if the participant spent at least 250 ms on-screen. We implemented this inclusion criterion to promote the inclusion of trials that were more likely to be representative of a participant’s true performance and exclude trials that likely include only spurious looks. We chose the minimum trial duration based on work from others [27] that has indicated that 200 ms is the minimum amount of time needed to characterize an orienting response. However, the mean time on-screen for each group was > 1000 ms, suggesting that for most participants, most trials included more than 250 ms of on-screen time. It is still important to consider why data loss may have occurred. Data loss in eye tracking reflects a quality control metric known as *robustness*. Robustness refers to the amount of eye tracking data recorded relative to data lost during recording. Data can be lost because the eye tracker is not able to detect the eye (e.g. because it failed to detect the corneal reflection), or because the participant is not looking at the screen (e.g. due to inattention). Infants have been reported to have less robust data than adults, and less robust data has been associated with longer response latencies [32]. In the present study we look at first feature fixated rather than first fixation, circumventing issues related to response latencies. More robust data has also been linked to longer average dwell times [32]. If the infant data in the present study is less robust than the data from school-age children, then this could have resulted in artificial differences in dwell times between the two groups. However, we computed and analyzed difference scores that reflect relative time on the different facial features within groups. This means that each group’s time on one feature, while proportional to their overall dwell time, is actually computed relative to that group’s time on the other feature(s). Within subjects, data loss can be assumed to be consistent and our groups were not directly compared in terms of absolute dwell time. This means that preference for a given feature within a group, and differences in preference between groups, should be unaffected by robustness.

Another methodological choice that warrants discussion is the size of the areas of interest. Specifically, the areas of interest are not matched for size such that the eyes region is twice the size of the mouth (i.e. each eye is matched in size to the mouth, but the eyes appear in pairs), and the other face parts are matched in size to the eyes on 60% of the trials, and matched to the mouth on 40% of the trials (i.e. the OFPs appear in pairs on 60% of the trials and appear alone on 40% of the trials). Thus, when the eyes are in competition with the mouth, participants may look at the eyes first because that region is larger than the mouth. We ultimately concluded that the current design of the stimuli is more ecologically valid than pairing single eyes with the mouth, or adjusting the data post-hoc (i.e. by area normalizing). We should also note that the eye region appeared above the center of the screen, though the mouth appeared below. While there is a possibility that dwell time on the eyes were driven by their location on the screen (i.e. a top-heavy stimulus), the placement of the facial features on the stimuli was chosen to reflect ecologically valid locations. However, the present results are likely unrelated to stimulus placement: a study investigating whether the location of the eyes on a face is what compels others to fixate them reported that the tendency to orient to eyes has more to do with the eyes themselves (i.e. their biological significance), than their positioning on the face [33]. Indeed, in the present study, the OFPs stimuli contained foreheads, which were even more top-heavy than the eyes, yet the preference for the eyes persisted.

The above caveats aside, the present study tested measurement invariance in a single eye-tracking task across a wide range of ages and ability levels. Our results suggest nominal age-related differences between the group of infants and school-aged children. Designing tasks that can be implemented across age, functioning level, and modality (e.g., eye tracking, EEG, fMRI) that provide meaningful individual differences that span the typical-atypical continuum is a major effort in our lab. Future work on measurement invariance/equivalence promises to inform long-term longitudinal studies that chart the natural history of autism spectrum disorders. Indeed, characterizing long-term stability and/or change in basic perceptual and attentional abilities promises to augment more traditional assessments of autistic symptoms or language level [2, 34]. If trajectories of development become a useful strategy to parse the heterogeneity of ASD, the utility of tasks suitable only for specific ages or functioning levels diminishes substantially. Further, many in the field are concerned with identifying biomarkers of treatment response, with a specific focus on eye-tracking and/or EEG [7]. Appreciating the fact that different interventions may be necessary for different ages, maintaining consistency in outcome biomarkers requires ensuring developmental invariance over time. While the task presented here did not reveal differences between children with and without autism, this in and of itself is interesting, especially considering other findings that suggest that individuals with autism have an abnormal tendency to fixate the mouth rather than the eyes (e.g. [11, 13, 14, 20, 29]). This task also serves as a proof-of-principle investigation of measurement invariance in a single eye-tracking task. More work is needed to develop a suite of tasks (eye-tracking and EEG tasks) that elicit meaningful individual differences between groups that could represent actionable metrics of treatment outcomes across a wide age range and broad functioning level.

## Supporting information

S1 File Data. Data from infants and school age children with and without autism. ReadMeData.pdf contains a description of data columns.
(ZIP)

S2 File Python script. Python script used to process eye tracking data. ReadMeScript.pdf provides instructions for use.
(ZIP)
