# Supplementary material for: Rapid face orienting in infants and school-age children with and without autism: Exploring measurement invariance in eye-tracking
Source: PLoS One. 2018 Aug 28;13(8):e0202875. doi: 10.1371/journal.pone.0202875 (PMC6112675; doi:10.1371/journal.pone.0202875)
Supplement: S1 File — Data from infants and school age children with and without autism. ReadMeData.pdf contains a description of data columns. (ZIP) [file pone.0202875.s001.zip › S1 File. Data/ReadMeData.pdf]

This information file accompanies the raw data from, Dalrymple, K.A., Wall, N. Spezio, M.L., Hazlett, H.C., Piven, J. & Elison, J.T. (2018). Rapid face orienting in infants and school-age children with and without autism: Exploring measurement invariance in eye-tracking. *PLOS ONE.*

Data are labeled by participant ID and task version (Bubbles 1, 2, or 3)
Data are organized into folders by:

- Collection location (UMN=University of Minnesota, UNC=University of North Carolina)
- Participant group (Infants vs. School age, and ASD vs. TYP within School age)
- Task version (Bubbles 1, 2, or 3)

Data files are in .csv format.

Table 1. Column header names and description of contents for raw data.

Column name	Description
001ParticipantName	Participant ID. All data have been de-identified.
002RecordingName	Recording name (no particular naming convention)
003MediaName	Name of stimulus that appeared on the screen at that time. evm = Eyes vs. Mouth; mvnff = Mouth vs. Other Face Parts; evnff = Eyes vs. Other Face Parts. M=Male, F=Female.
004RecordingTimestamp	Time from beginning of recording in ms.
005GazeEventType	Fixation vs. saccade vs. unclassified. Because raw data was exported (i.e. no fixation filter was applied), all detected events are classified as fixations.
006GazeEventDuration	Duration of gaze event in ms. Because raw data was exported (i.e. no fixation filter was applied), all detected events were ~3ms for data collected on the Tobii X300 eye tracker and ~8ms for data collected on the Tobii X120 eye tracker.
All remaining columns: AOIs	Each column labeled "AOI[stimulusname_aoi]Hit" contains 1s and 0s to indicate whether the eyes were detected on a given Area Of Interest. _le=left eye, _re=right eye, _m=mouth, _nff1= other face part 1, _nff2=other face part 2, _x= fixation, _screen= fixations that land anywhere on the screen. Note 1: not all AOIs apply to all stimuli, e.g. there is no left eye or right eye for a "mouth vs. other face parts" stimulus. Note 2: if a fixation lands on an AOI (e.g. left eye), there will be a 1 in the column for that AOI (left eye) and in the column for "screen", but this is only one fixation. That is, all fixations on particular AOIs will also be recorded in the "screen" column, but fixations that land on the screen may not necessarily appear in another AOI column if the participant fixated the background.
