# Supplementary material for: Rapid face orienting in infants and school-age children with and without autism: Exploring measurement invariance in eye-tracking
Source: PLoS One. 2018 Aug 28;13(8):e0202875. doi: 10.1371/journal.pone.0202875 (PMC6112675; doi:10.1371/journal.pone.0202875)
Supplement: S2 File — Python script used to process eye tracking data. ReadMeScript.pdf provides instructions for use. (ZIP) [file pone.0202875.s002.zip › S2 File. Python Script/ReadMeScript.pdf]

User Notes for Bubbles.py

Script was written by Marie D. Manner, for Dalrymple, K.A., Wall, N. Spezio, M.L., Hazlett, H.C., Piven, J. & Elison, J.T. (2018). Rapid face orienting in infants and school-age children with and without autism: Exploring measurement invariance in eye-tracking. *PLOS ONE.*

Background:
Bubbles.py was written to calculate duration of dwell time and time to fixate the different AOIs in the "bubbles" task described in Dalrymple et al. The script is provided as is-- the authors will not provide technical support.

Requirements:
Download Python from www.python.org. Requires Version 3.0 or above. Version 3.3 recommended.

Implementation:

1. Collect eye movement data from participants.

2. Set fixation filters to Raw Data

3. Output data columns listed in Table 1 in the order listed (see Figure 1).

4. Save as .csv file, one recording/participant per file.

4. Put all .csv files in one folder and save on the desktop.

5. Open Bubbles.py by double clicking on the file.

6. Select script window, Run→ Run Module (F5 on Mac).

7. When prompted, select the folder that contains the .csv data files.

8. Output for .csv files will appear in the folder that contains the original .csv files. Output files will be named DataFileName_output.csv, where "DataFileName" is the name of each individual data file provided.

Table 1. Input (See Fig 1 for sample input)

Column	Column Header (row 1)	Description
A	ParticipantName	Participant ID
B	RecordingDate	Date of recording
C	MediaName	Name of stimuli.
D	RecordingTimestamp	Recording time, in ms.
E	GazeEventType	Fixation, saccade, or unclassified
F	GazeEventDuration	Duration of the event, in ms.
G	AOI 1.1	AOI 1 for stimulus 1.
H	AOI 1.2	AOI 2 for stimulus 1.
.	.	.
.	.	.
.	.	.
X	AOI 1.X	An AOI that covers the entire screen for stimulus 1. This “background AOI” should always be the last AOI listed for the stimulus.
Y	AOI 2.1	AOI 1 for stimulus 2
Z	AOI 2.2	AOI 2 for stimulus 2

Table 2. Output explained. See Fig 2 for sample output.

Column	Description
Participant………………………………………………..	Participant ID.
Stimuli…………………………….....………………..	Stimulus that appeared on the screen on that trial.
LeftEye ………….…………………………….………	Total dwell time duration on LeftEye in ms, if this AOI was present. Value = 0 if this AOI was not present.
RightEye ……….………………………….....……….	Total dwell time on RightEye in ms, if this AOI was present. Value = 0 if this AOI was not present.
Mouth ………………………………………………...	Total dwell time on Mouth in ms, if this AOI was present. Value = 0 if this AOI was not present.
OFP1 ……………………………………………….	Total dwell time on Other Face Part 1 in ms, if this AOI was present. Value = 0 if this AOI was not present.
OFP2…………………………………………………	Total dwell time on Other Face Part 2 in ms, if this AOI was present. Value = 0 if this AOI was not present.
Fix* ………………………………………………..	Total dwell time on fixation shape in ms, if this AOI was present. Value = 0 if this AOI was not present.
Background …………………………………….	Total dwell time on Background, in ms. This total is the accumulated dwell time from when participant gaze is on screen, but not on any defined AOI.
Screen…………………………………………………	Total dwell time on screen, in ms. This total is the accumulated dwell time from when participant gaze is on screen, regardless of where gaze lands on the screen.

TimetoLE………………………………………	Time from beginning of trial until gaze lands on Left Eye, in ms. Value = 9999 if gaze never lands on this AOI, or if this AOI doesn't exist.
TimetoRE………………………………………	Time from beginning of trial until gaze lands on Right Eye, in ms. Value = 9999 if gaze never lands on this AOI, or if this AOI doesn't exist.
TimetoMouth………………………………………	Time from beginning of trial until gaze lands on Mouth, in ms. Value = 9999 if gaze never lands on this AOI, or if this AOI doesn't exist.
TimetoOFP1………………………………………	Time from beginning of trial until gaze lands on Other Face Part 1, in ms. Value = 9999 if gaze never lands on this AOI, or if this AOI doesn't exist.
TimetoOFP2………………………………………	Time from beginning of trial until gaze lands on Other Face Part2, in ms. Value = 9999 if gaze never lands on this AOI, or if this AOI doesn't exist.
TimeToFix*………………………………………	Time from beginning of trial until gaze lands on fixation mark, in ms. Value = 9999 if gaze never lands on fixation mark.
Total Trial………………………………………	Total trial duration, in ms..

* For our analysis, we made an AOI for where the fixation shape was just prior to the stimulus presentation.

Figure 1. Sample input.

	001ParticipantName	002RecordingName	003MediaName	004RecordingTimestamp	005GazeEventType	006GazeEventDuration	AOI[01.1_o1_f4_evm_0_l_le]Hit	AOI[01.2_o1_f4_evm_0_l_re]Hit	AOI[01.3_o1_f4_evm_0_l_r
2	JE000015_03_03	Rec 67_01	o1_m16_evm_0_r.jpg	5296	Fixation	3			
3	JE000015_03_03	Rec 67_01	o1_m16_evm_0_r.jpg	5300	Fixation	3			
4	JE000015_03_03	Rec 67_01	o1_m16_evm_0_r.jpg	5303	Fixation	3			
5	JE000015_03_03	Rec 67_01	o1_m16_evm_0_r.jpg	5306	Fixation	3			
6	JE000015_03_03	Rec 67_01	o1_m16_evm_0_r.jpg	5310	Fixation	3			
7	JE000015_03_03	Rec 67_01	o1_m16_evm_0_r.jpg	5313	Fixation	3			
8	JE000015_03_03	Rec 67_01	o1_m16_evm_0_r.jpg	5316	Fixation	3			
9	JE000015_03_03	Rec 67_01	o1_m16_evm_0_r.jpg	5320	Fixation	3			
10	JE000015_03_03	Rec 67_01	o1_m16_evm_0_r.jpg	5323	Fixation	3			
11	JE000015_03_03	Rec 67_01	o1_m16_evm_0_r.jpg	5326	Fixation	3			
12	JE000015_03_03	Rec 67_01	o1_m16_evm_0_r.jpg	5330	Fixation	3			
13	JE000015_03_03	Rec 67_01	o1_m16_evm_0_r.jpg	5333	Fixation	3			
14	JE000015_03_03	Rec 67_01	o1_m16_evm_0_r.jpg	5336	Fixation	3			
15	JE000015_03_03	Rec 67_01	o1_m16_evm_0_r.jpg	5340	Fixation	3			
16	JE000015_03_03	Rec 67_01	o1_m16_evm_0_r.jpg	5343	Fixation	3			
17	JE000015_03_03	Rec 67_01	o1_m16_evm_0_r.jpg	5346	Fixation	3			
18	JE000015_03_03	Rec 67_01	o1_m16_evm_0_r.jpg	5350	Fixation	3			
19	JE000015_03_03	Rec 67_01	o1_m16_evm_0_r.jpg	5353	Fixation	3			
20	JE000015_03_03	Rec 67_01	o1_m16_evm_0_r.jpg	5356	Fixation	3			
21	JE000015_03_03	Rec 67_01	o1_m16_evm_0_r.jpg	5360	Fixation	3			
22	JE000015_03_03	Rec 67_01	o1_m16_evm_0_r.jpg	5363	Fixation	3			
23	JE000015_03_03	Rec 67_01	o1_m16_evm_0_r.jpg	5366	Fixation	3			
24	JE000015_03_03	Rec 67_01	o1_m16_evm_0_r.jpg	5369	Fixation	3			
25	JE000015_03_03	Rec 67_01	o1_m16_evm_0_r.jpg	5373	Fixation	3			
26	JE000015_03_03	Rec 67_01	o1_m16_evm_0_r.jpg	5376	Fixation	3			
27	JE000015_03_03	Rec 67_01	o1_m16_evm_0_r.jpg	5380	Fixation	3			
28	JE000015_03_03	Rec 67_01	o1_m16_evm_0_r.jpg	5383	Fixation	3			
29	JE000015_03_03	Rec 67_01	o1_m16_evm_0_r.jpg	5386	Fixation	3			
30	JE000015_03_03	Rec 67_01	o1_m16_evm_0_r.jpg	5390	Fixation	3			
31	JE000015_03_03	Rec 67_01	o1_m16_evm_0_r.jpg	5393	Fixation	3			
32	JE000015_03_03	Rec 67_01	o1_m16_evm_0_r.jpg	5396	Fixation	3			
33	JE000015_03_03	Rec 67_01	o1_m16_evm_0_r.jpg	5399	Fixation	3			
34	JE000015_03_03	Rec 67_01	o1_m16_evm_0_r.jpg	5403	Fixation	3			
35	JE000015_03_03	Rec 67_01	o1_m16_evm_0_r.jpg	5406	Fixation	3			
36	JE000015_03_03	Rec 67_01	o1_m16_evm_0_r.jpg	5410	Fixation	3			
37	JE000015_03_03	Rec 67_01	o1_m16_evm_0_r.jpg	5413	Fixation	3			
38	JE000015_03_03	Rec 67_01	o1_m16_evm_0_r.jpg	5416	Fixation	3			
39	JE000015_03_03	Rec 67_01	o1_m16_evm_0_r.jpg	5420	Fixation	3			
40	JE000015_03_03	Rec 67_01	o1_m16_evm_0_r.jpg	5423	Fixation	3			

Figure 2. Sample output.

	A	B	C	D	E	F	G	H	I	J	K	L	M	N	O	P	Q
1	Participant	Stimuli	LeftEye	RightEye	Mouth	OFP1	OFP2	Fix	Background	Screen	TimetoLE	TimetoRE	TimetoMout	TimetoOFP1	TimetoOFP2	TimeToFix	Total Trial
2	JE000015_03	o1_f10_mvn	0	0	0	171	0	44	409	624	9999	9999	9999	57	9999	77	2023
3	JE000015_03	o1_f11_evnf	575	730	0	189	0	135	397	2026	250	816	9999	1823	9999	0	2023
4	JE000015_03	o1_f12_evnf	0	0	0	0	0	0	0	0	9999	9999	9999	9999	9999	9999	2029
5	JE000015_03	o1_f14_evnf	0	120	0	225	0	9	222	576	9999	1910	9999	1637	9999	1650	2027
6	JE000015_03	o1_f14_evnf	0	0	0	0	0	0	0	0	9999	9999	9999	9999	9999	9999	2026
7	JE000015_03	o1_f15_evnf	0	0	0	0	0	0	0	0	9999	9999	9999	9999	9999	9999	2023
8	JE000015_03	o1_f1_evm_	806	603	0	0	0	0	347	1756	540	1423	9999	9999	9999	9999	2023
9	JE000015_03	o1_f2_evm_	363	747	0	0	0	0	362	1472	834	1237	9999	9999	9999	9999	2023
10	JE000015_03	o1_f3_evm_	980	0	0	0	0	182	451	1613	293	9999	9999	9999	9999	0	2019
11	JE000015_03	o1_f4_evm_	0	412	46	0	0	27	265	750	9999	1527	1394	9999	9999	1490	2017
12	JE000015_03	o1_f5_evm_	0	0	0	0	0	0	0	0	9999	9999	9999	9999	9999	9999	2023
13	JE000015_03	o1_f6_mvnff	0	0	976	0	0	0	848	1824	9999	9999	450	9999	9999	9999	2030
14	JE000015_03	o1_f7_mvnff	0	0	740	0	26	69	948	1783	9999	9999	773	9999	443	500	2029
15	JE000015_03	o1_f8_mvnff	0	0	295	1353	0	32	342	2022	9999	9999	1603	50	9999	0	2019
16	JE000015_03	o1_f9_mvnff	0	0	0	3	0	0	214	217	9999	9999	9999	190	9999	9999	2030
17	JE000015_03	o1_m16_evn	574	904	0	0	0	56	492	2026	333	1037	9999	9999	9999	7	2023
18	JE000015_03	o1_m17_evn	6	540	546	0	0	159	340	1591	1847	400	1250	9999	9999	130	2027
19	JE000015_03	o1_m18_evn	998	0	0	0	0	108	434	1540	244	9999	9999	9999	9999	10	2030
20	JE000015_03	o1_m19_evn	0	743	0	0	0	33	353	1129	9999	229	9999	9999	9999	0	2023
21	JE000015_03	o1_m20_evn	0	0	0	0	0	0	0	0	9999	9999	9999	9999	9999	9999	2020
22	JE000015_03	o1_m21_evn	190	747	0	530	0	13	83	1563	497	693	9999	1353	9999	1330	2023
23	JE000015_03	o1_m22_evn	363	921	0	0	0	16	393	1693	356	736	9999	9999	9999	0	2023
24	JE000015_03	o1_m23_evn	621	1073	0	0	0	30	239	1963	233	860	9999	9999	9999	167	2030
25	JE000015_03	o1_m24_evn	0	0	0	0	0	0	0	0	9999	9999	9999	9999	9999	9999	2033
26	JE000015_03	o1_m25_evn	0	0	0	0	0	0	0	0	9999	9999	9999	9999	9999	9999	2026
27	JE000015_03	o1_m26_mv	0	0	0	0	390	0	600	990	9999	9999	9999	9999	1380	9999	2010
28	JE000015_03	o1_m27_mv	0	0	270	165	0	0	707	1142	9999	9999	880	1256	9999	9999	2019
29	JE000015_03	o1_m28_mv	0	0	133	993	0	156	491	1773	9999	9999	963	13	9999	0	2019
30	JE000015_03	o1_m29_mv	0	0	0	0	0	0	0	0	9999	9999	9999	9999	9999	9999	2034
31	JE000015_03	o1_m30_mv	0	0	0	0	0	0	0	0	9999	9999	9999	9999	9999	9999	2020
32	JE000015_03	zAVERAGE	182.53	251.33	100.2	120.97	13.87	35.63	297.9	1002.43	623.67	0	908	628	911.5	78.5	2024.13
